# Microbes as manipulators of egg size and developmental evolution

**DOI:** 10.1128/mbio.03655-24

**Published:** 2025-04-17

**Authors:** Matthew C. Kustra, Tyler J. Carrier

**Affiliations:** 1 Department of Ecology and Evolutionary Biology, University of California, Santa Cruz, California, USA; 2 Department of Integrative Biology, University of California, Berkeley, California, USA; 3 Miller Institute for Basic Research in Science, University of California, Berkeley, California, USA; 4 GEOMAR Helmholtz Centre for Ocean Research, Kiel, Germany; 5 Zoological Institute, Kiel University, Kiel, Germany; Max Planck Institute for Marine Microbiology, Bremen, Germany

**Keywords:** evolution, marine invertebrates, microbiome, symbiosis, *Wolbachia*, mathematical model

## Abstract

Marine invertebrates mainly reproduce by energy-poor eggs that develop into feeding larvae or energy-rich eggs that develop into non-feeding larvae. Evolutionary transitions between these developmental modes have been studied in detail, yet the evolutionary factor(s) responsible for these switches remains elusive. Here, we use theoretical models to support the premise that microbes with the capacity to manipulate host reproduction may be one possible factor. Our model predicts that microbial manipulators could create a sperm-limited environment that selects for larger eggs by shifting the host’s sex ratio toward female dominance and, as a result, drive an evolutionary transition in the developmental mode for marine invertebrates. The loss of a microbial manipulator could then recover the ancestral egg size and developmental mode. We also suggest more than a dozen genera of marine invertebrates from throughout the world’s oceans that fit the framework of a microbe-induced evolutionary transition between these predominant developmental modes. We anticipate that microbial manipulators have a yet-to-be-appreciated influence on the developmental evolution of marine invertebrates. We find it paramount to understand whether evolutionary transitions in developmental mode occur with and without microbial manipulators as well as whether the underlying mechanisms of these manipulations are convergent with terrestrial systems.

**IMPORTANCE:**

Microbes that manipulate animal reproduction are widespread on land, and their evolutionary influence is widely acknowledged. Relatives of these manipulators are increasingly found in the ocean, but uniquely with taxa that recently underwent a transition in developmental evolution from feeding to non-feeding larvae. Here, we present theoretical models supporting that microbial manipulators could create a sperm-limited environment that selects for larger eggs by shifting the host’s sex ratio toward female dominance and, as a result, drive an evolutionary transition in the developmental mode for free-spawning marine invertebrates. This theoretical model provides a complementary viewpoint to the theory regarding the evolutionary process that marine invertebrates undergo to transition between developmental modes as well as a fruitful opportunity to compare with terrestrial systems.

## INTRODUCTION

The most common reproductive strategy among marine invertebrates involves producing a high number of small, energy-poor eggs that develop into feeding larvae (i.e., planktotrophy) (1–6). This stability has occasionally been disrupted over the last several hundred million years by a rapid switch in developmental mode (1–6). Species with the derived reproductive strategy produce fewer large, energy-rich eggs and larvae that undergo metamorphosis without feeding (i.e., lecithotrophy) (1–6). Evolutionary transitions in developmental and reproductive modes have occurred in most major animal phyla, are primarily unidirectional, and intermediates between planktotrophy and lecithotrophy are rare (1–3, 5–7). The factor(s) responsible for driving evolutionary transitions in reproductive strategy and developmental mode remain elusive despite having been studied in detail at the cellular, genomic, and molecular levels (6, 8–14).

Microbes may be one such factor, as they are functionally beneficial to animal reproduction and have evolved mechanisms to override components of this host program (15–17). Microbial manipulators (e.g., *Wolbachia*) are often inherited through the cytoplasm of the egg and manipulate the host to favor their transmission (e.g., by sex ratio distortion toward female dominance) (15, 16). Hosts affected by reproductive manipulators that induce cytoplasmic incompatibility (microbe-containing males are incompatible with aposymbiotic females), feminization (the conversion of genetic males to females), or male killing (microbe-induced male mortality) are gonochoric, diverse, and particularly common in terrestrial arthropods and nematodes (15, 16, 18, 19). Microbes that are phylogenetically related to and exhibit shared genomic features with terrestrial reproductive manipulators are increasingly found in the ocean (20–22). These microbes are associated with major marine invertebrate phyla (20–22), with the sea urchin *Heliocidaris* being the most well-defined example (20, 23).

*Heliocidaris* is one of the most comprehensively studied systems that document the processes marine invertebrates undergo following an evolutionary transition between major developmental modes (1, 13, 14, 20, 23). A speciation event ~5 million years ago resulted in sister species with alternative reproductive strategies. This has been presumed to have taken place along the Eastern coast of Australia, where both species can still be found to overlap geographically (24). The ancestral *H. tuberculata* is planktotrophic and the derived *H. erythrogramma* is lecithotrophic (6, 25). Typical of planktotrophs, *H. tuberculata* develops from small eggs into feeding larvae that disperse for several weeks, while *H. erythrogramma* develops from eggs ~53× to 86× the volume of *H. tuberculata* eggs into non-feeding larvae that lack the morphological structures for feeding (e.g., a functional gut) and remain in the water column for only 5 days (6, 26). Moreover, this evolutionary transition in developmental mode corresponded with a rewiring of the gene regulatory network (8, 13), reorganization of cell fates (14), and modification to gametogenesis (27).

The lecithotrophic *H. erythrogramma* acquired a cytoplasmically inherited Rickettsiales—*Echinorickettsia raffii*—in this transition in developmental mode that is most closely related to *Wolbachia pipientis* (20). The genome of *E. raffii* suggests that this bacterium is a nutritional mutualist and a reproductive manipulator (20). In the former, the host is hypothesized to benefit from symbiont-derived essential amino acids to enhance growth, as is observed in terrestrial systems (28, 29). In the latter, this bacterium is hypothesized to use effector proteins via a Type IV secretion system to influence the rate of male mortality and modulate fertilization (20). Supporting the hypothesis that *E. raffii* manipulates host reproduction, populations of *H. erythrogramma* in Sydney Harbor and Tasmania are disproportionately female (20, 30). Moreover, the youngest reproductive individuals in Sydney Harbor do not deviate from a 1:1 female-to-male ratio, while the largest individuals have a 4:1 female-to-male ratio (20). There is currently no evidence that *E. raffii* is also an endosymbiont of the planktotrophic *H. tuberculata* (20), indicating potential differences in whether and how reproductive manipulators may influence and spread these two developmental strategies.

The identification of a suspected reproductive manipulator in the sea urchin *Heliocidaris* has spurred a theoretical test for whether microbial manipulators can use the vastly different reproductive strategies of marine invertebrates to spread among their oceanic populations (23). The primary predicted difference between terrestrial and marine reproductive manipulators is that there appears to be an unbalanced influence on the two predominant developmental modes found in the ocean (23). Microbial manipulators and their ability to distort host sex ratio toward female dominance are thought to limit the reproductive success of ancestral planktotrophs (due to lower fertilization caused by a sperm limitation), while enhancing offspring production and population size for the derived lecithotrophs (23). This could create a gradient where reproductive manipulators may serve as a selective agent to induce an evolutionary transition between these major reproductive strategies.

We tested this hypothesis using theoretical models to simulate the eco-evolutionary dynamics of egg size (diameter, μm)—a proxy for developmental mode (4, 6, 31)—for marine invertebrates that free-spawn and are gonochoric following a novel association with a microbial manipulator. Specifically, we integrated evolutionary processes into the ecology-focused theoretical model that was established to assess the population-level dynamics of reproductive manipulators in the ocean (23). The eco-evolutionary theoretical model presented here predicts that microbial manipulators could create a sperm-limited environment that selects for larger eggs by shifting the host’s sex ratio toward female dominance and, as a result, serve as a driver of an evolutionary transition in the developmental mode of marine invertebrates. We also suggest more than a dozen genera of marine invertebrates that fit the framework of a microbe-induced evolutionary transition in developmental mode.

## RESULTS

### Microbial manipulators alter egg size

**Fig 1:**
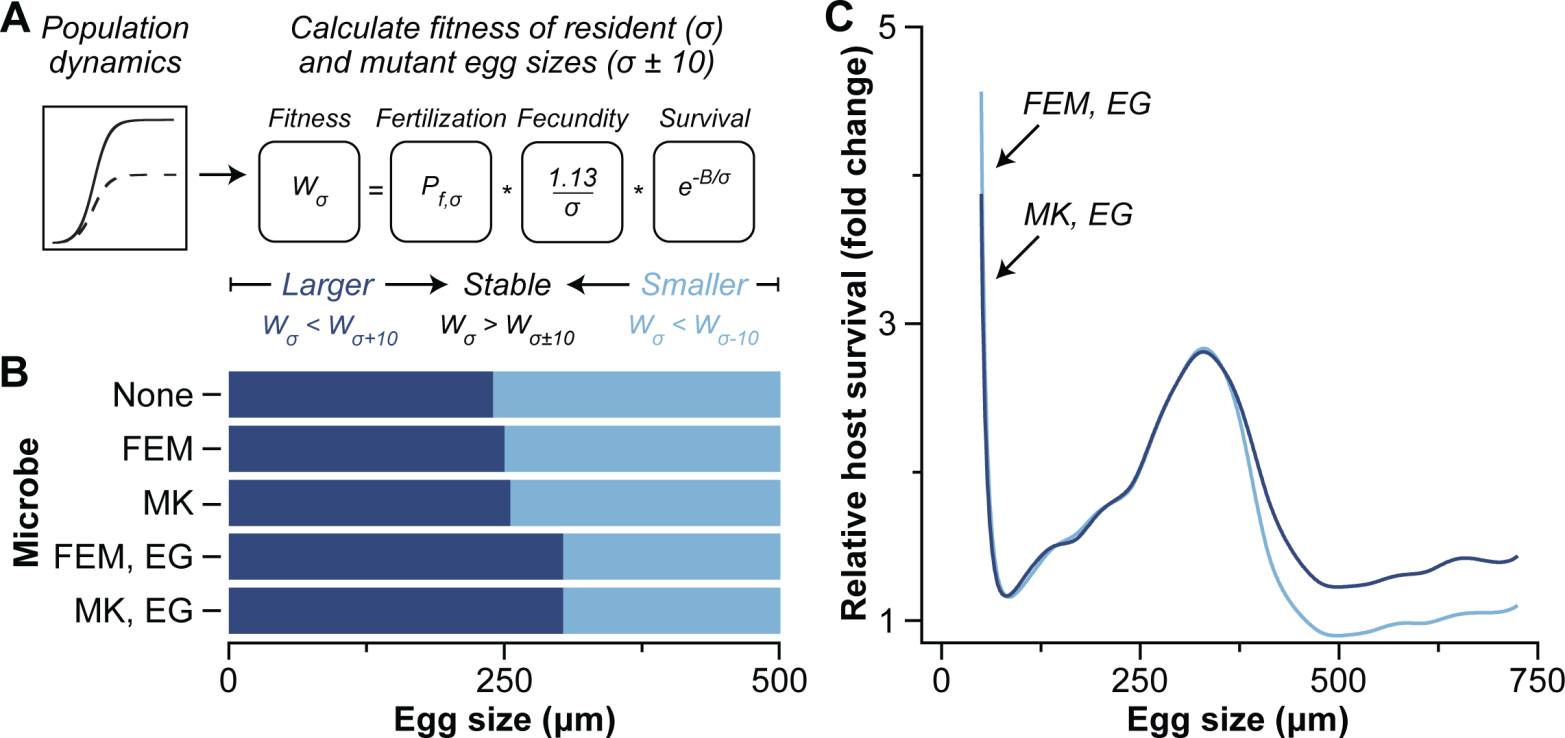
Microbial manipulators alter egg size. (**A**) A grid-based invasion analysis was used to determine whether egg size evolves following a novel association with a microbe that manipulates host reproduction by feminization (FEM) or male killing (MK) and can compensate for host fitness by supplementing host nutrition (EG). The fitness of an individual producing the resident egg size (σ) was calculated and compared to rare mutants that produced either slightly smaller or larger egg sizes (σ ± 10 µm). Stable points were identified by the resident egg size having greater fitness (*W;* i.e*.*, the number of offspring based on the probability of fertilization [*P*], fecundity, and survival) than either mutant. (**B**) A representative example of the evolutionary direction of egg size with no microbe, FEM (*r =* 0.845), MK (*m_k_* = 0.84), and FEM or MK and EG (*g* = 1.5) at a *B* parameter value of 1,000. (**C**) A representative example of the relative host survival (i.e., fold change) following a novel association with a microbe that manipulates by FEM or MK and EG was determined using the stable egg size across *B* values and back-calculating for survival (i.e., fitness) relative to a population that did not associate with a microbial manipulator. MK, FEM, and EG rates are the same as panel B.

We used a deterministic grid-based invasion analysis to test whether egg size evolves following a novel association with a microbe that manipulates host reproduction by feminization (*r*, 0.5–0.995 at increments of 0.015) or male killing (*m_k_*, 0–0.99 at increments of 0.03) and can compensate host fitness by supplementing host nutrition (*g*, 0–3.0 at increments of 0.1) (16, 32). We simulated the population dynamics of free-spawning marine invertebrates across a range of potential egg size-offspring survival relationships that were derived from Vance (33, 34), with the shape of this relationship being determined by the *B* parameter (100–3,000 at increments of 100; Fig. S1). Larger *B* values indicate ecological scenarios that increase the importance of larger egg size for offspring survival (Fig. S1). We assume that all resident females produce eggs of the same size, that fecundity is inversely proportional to egg volume (i.e., maternal investment), and that individuals with larger eggs have an increased probability of fertilization and survival (1, 4, 33). Once the populations stabilized, we calculated the fitness of resident (σ) and mutant (σ ± 10 µm) egg sizes and then recorded the direction of evolution at each point on the grid (50 μm to 1,500 µm at increments of 10 µm; Fig. 1A).

We observed that the presence of a microbial manipulator altered the egg size of free-spawning marine invertebrates (Fig. 1B). The magnitude of egg size evolution depended on the interaction between manipulation, enhanced growth, and the underlying ecology (i.e., the *B* parameter) (Fig. S1 to S4). Male killing and feminization alone resulted in a very slight increase in egg size across most ecological scenarios, while the combination of manipulation and enhanced growth led to the evolution of much larger eggs (Fig. 1; Fig. S1 to S4). The latter, however, was dependent on the underlying ecology. Stable egg size was larger for free-spawning marine invertebrates associated with a microbe that was manipulated by male killing and enhanced growth compared to no manipulation across all *B* values. A similar pattern was observed for a microbe that was manipulated by feminization and enhanced growth until a *B* value of >2,100, after which egg sizes were similar with and without a microbial manipulator (Fig. S1 to S4).

Evolutionary shifts in stable egg size also led to notable differences in the relative fitness (i.e., survival) of populations of free-spawning marine invertebrates due to the microbial manipulator (Fig. 1C). In this representative example, the relative fitness of a free-spawning marine invertebrate with (as compared to without) a microbial manipulator was highest (i.e., between ~3.9× and ~4.6×) for populations that had egg sizes less than 100 µm. Relative fitness then increased to a second maxima of ~3.3× at an egg size of ~330 µm for populations with a manipulator that induced feminization or male killing and enhanced growth (Fig. 1C). The magnitude of this maxima and the egg size at which this peak occurred increased with the rate of manipulation for both male killing and feminization (Fig. S2). Relative fitness was then most similar to not associating with a manipulator that induced either feminization or male killing and enhanced growth at an egg size of ~500 µm, after which the relative fitness with a microbe increased slightly thereafter (Fig. 1C). As such, both manipulators had a similar peak, but the relative fitness of egg sizes past this peak was higher and broader for those that induced male killing and enhanced growth (Fig. 1C). Interestingly, these two maxima in relative fitness coincide with the egg sizes for planktotrophy and lecithotrophy and, thus, are consistent with the theoretical consensus of developmental evolution and reproductive strategies of marine invertebrates that is supported by a wealth of natural history data (1, 6, 9, 31, 33, 35, 36). Therefore, it is implied that microbes could be an inductive factor along this fitness gradient and of an evolutionary transition in developmental mode.

### Microbial manipulators induce an evolutionary transition in developmental mode

The grid-based invasion analysis implies that the increase in egg size due to a novel association with a microbial manipulator could induce a transition from the ancestral planktotroph to the derived lecithotroph (Fig. 1). This approach has limitations because it assumes that egg size does not vary within a population and that the underlying ecological and evolutionary processes are separate. This model is also unable to assess the relative speed of an evolutionary transition and the sequence of events leading to a switch in developmental mode. Therefore, we established a deterministic quantitative genetic population model that relaxes these assumptions, allowing us to directly test whether a novel association with a microbial manipulator could serve as the selective pressure of egg size evolution that, in turn, induces a transition in developmental mode.

This quantitative genetic population model assumes that egg size is a normally distributed trait in a population ($\bar{x}$ ± 10 standard deviation of 9 µm) and calculates the egg size-based fitness of females using the same equations as the invasion grid approach (Fig. 1A). The change in mean egg size at each generation was calculated using the quantitative genetic breeder’s equation. Constant variation was assumed for each generation, and iterations were performed until the mean egg size reached a stable point. Microbes are also known to interact with the molecular machinery used in yolk production (37), influence maternal provisioning and offspring fitness (38, 39), and their abundance (i.e., titer) can scale allometrically with offspring size (40–42). Therefore, we established the *V* parameter to account for the possibility that microbial presence and abundance influence the relationship between egg size and offspring survival (i.e., the effective *B* parameter) (Fig. S5). As such, the *V* parameter can be used to alter the proportional influence that microbial abundance—a proxy for the functional capacity of that microbial population—has on the offspring survival-egg size relationship, with smaller *V* values representing a greater influence on that relationship (Fig. S5).

**Fig 2:**
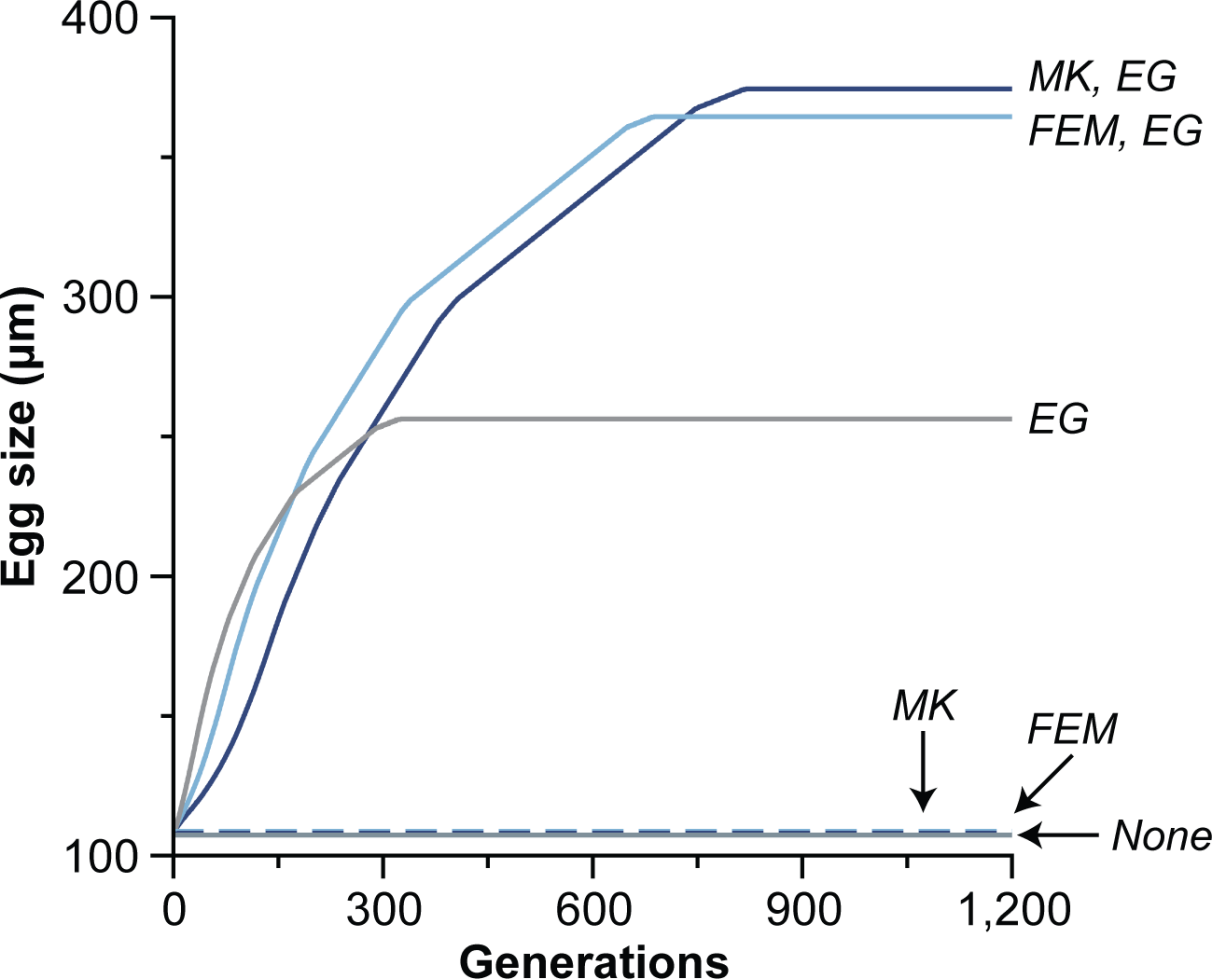
Microbial manipulators induce an evolutionary transition in developmental mode. If a free-spawning marine invertebrate is associated with a microbe that has the capacity for feminization (FEM; dashed light blue) or male killing (MK; dashed dark blue), then egg size would often increase slightly. If this microbe can enhance growth (EG; light gray), then egg size usually does not increase enough to induce a transition in developmental mode. If that microbe can manipulate and enhance growth (solid blue lines), then egg size most often would increase enough (i.e., >300 µm) that the host undergoes an evolutionary transition in developmental mode from the ancestral planktotroph to the derived lecithotrophy (6, 31). This representative example uses the following parameters: *m_k_* = 0.85, *r* = 0.85, *g* = 0.5, *B* = 250, and *V* = 250.

We introduced several types of microbial manipulators to a population of free-spawning marine invertebrates that had a stable egg size well within range of a typical planktotroph (i.e., 107 µm; Fig. 2) (1, 4, 6, 31). If the microbial manipulator was only capable of feminization (*r*, 0.85) or male killing (*m_k_*, 0.85), then egg size often increased only a few micrometers (Fig. 2). If the microbe was only capable of enhanced growth (*g*, 0.5; *v*, 250), then egg size increased significantly but usually not enough to induce a switch in developmental mode (Fig. 2). If the microbe was capable of manipulation and enhanced growth, then the increase in egg size would often be sufficient (i.e., >300 µm) to induce an evolutionary transition from planktotrophy to lecithotrophy (Fig. 2). In this representative example, egg size for these populations became stable after ~690 to ~820 generations, while the distortion in sex ratio caused by the microbial manipulator fixated 32.8× to 45.9× more quickly (Fig. 2; Fig. S6). If this microbe was lost following an evolutionary transition in developmental mode, then the initial egg size can nearly be recovered and does so 3.4× to 3.9× more quickly than the initial association (Fig. S6).

### Conditions of a microbe-induced evolutionary transition in developmental mode

The dynamics described above is an example from a representative set of parameters that demonstrates how a microbial manipulator could induce an evolutionary transition in developmental mode via egg size evolution in a free-spawning marine invertebrate (Fig. 2). This, however, may not encompass the spectrum of interactions that can be observed between marine invertebrates and microbial manipulators. Therefore, we assessed the conditions under which a microbe-induced evolutionary transition in developmental mode is observed. We ran our deterministic quantitative genetic population model across all possible combinations of *B* values (250–750 at increments of 10), *V* values (250–750 at increments of 10), and male killing (*m_k_*, 0–0.98 at increments of 0.02) or feminization (*r*, 0.5–0.99 at increments of 0.01) rates. Initial analyses showed that the presence, not magnitude, of enhanced growth was important and, thus, we only explored no (*g* = 0) or small (*g* = 0.5) enhanced growth (Fig. S7). Moreover, the *V* parameter was ignored when there was no enhanced growth (*g* = 0) because it is another aspect of enhanced growth.

**Fig 3:**
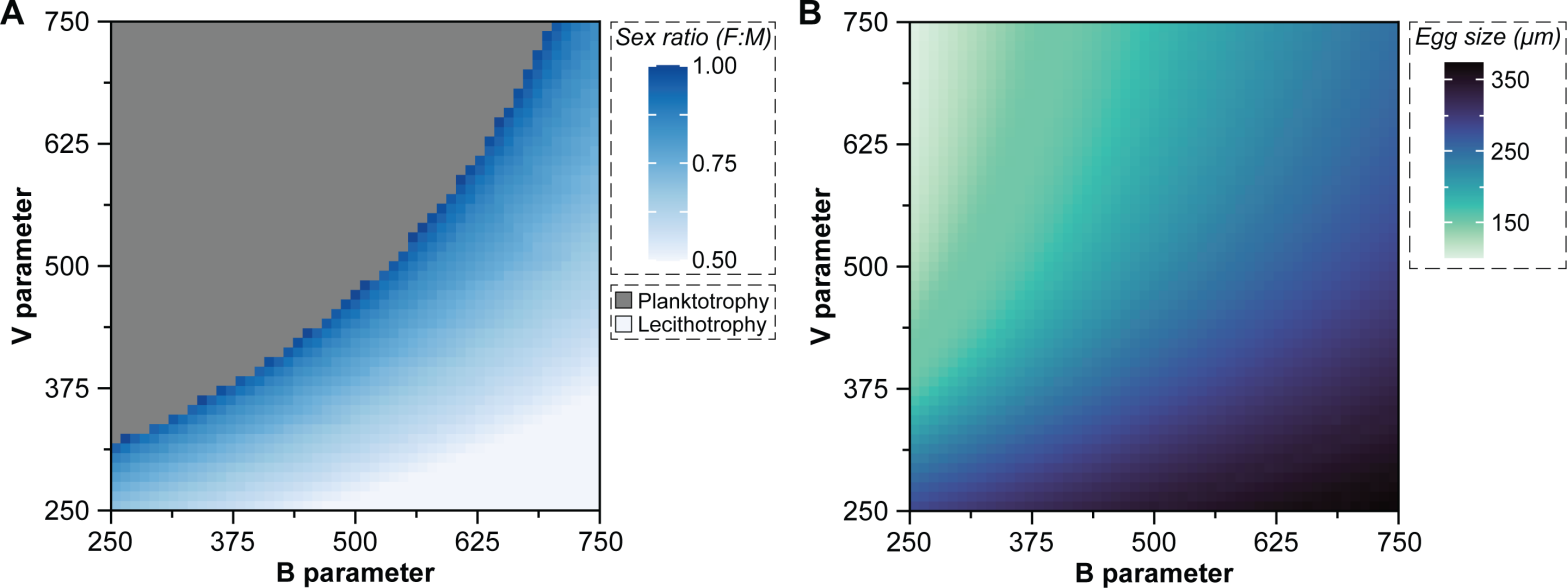
Conditions of a microbe-induced evolutionary transition in developmental mode. The conditions for a microbe-mediated evolutionary transition in developmental mode depend on the ecological conditions of the host (i.e., *B* parameter) as well as the manipulating capability of the microbe (i.e., *V* parameter). These factors create a gradient in the sex ratio (**A**) needed to induce a transition in developmental mode (i.e., at 300 µm) as well as a gradient in the stable egg sizes that follow this transition (**B**), with the mean egg size for each combination presented here. No transition may also be observed when ecology heavily favors planktotrophy and the manipulating capability of the microbe is weak (gray, top left of panel A). When ecology favors larger egg sizes, only enhanced growth is needed for a transition to occur and no manipulation in sex ratio (light blue, bottom right of panel A). The microbe here represents male killing and enhanced growth, but a nearly identical pattern is also observed for a microbe that has the capacity for feminization and enhanced growth (see Fig. S8).

Microbe-induced evolutionary transitions in developmental mode due to egg size evolution were observed across a wide range of conditions (Fig. 3; Fig. S8). These evolutionary transitions were more likely to occur when the microbe was capable of manipulation and enhanced growth (31.5% for feminization and 25.5% for male killing) than only enhanced growth (6.6%) or manipulation (0% for feminization or male killing) (Fig. 3; Fig. S8). Moreover, a more extreme microbe-induced sex ratio distortion is generally needed for an evolutionary transition in developmental mode when ecology favors the ancestral planktotroph (i.e., as the *B* parameter decreased and the *V* parameter increased) (Fig. 3; Fig. S8). A microbe-induced evolutionary transition in developmental mode also led to a wide range of stable egg sizes, which increased exponentially as the population was manipulated toward female dominance (Fig. S9 and S10). Microbes capable of feminization or male killing and enhanced growth increased to an average egg size of 364 µm (± 58 µm, standard deviation) and 353 µm (± 50, standard deviation) and a maximal egg size of 624 µm and 583 µm, respectively (Fig. 3B; Fig. S11). This equates to an average increase in relative egg volume (µm^3^) of 8.7× and 7.7× and a maximal increase in relative egg volume of 59.3× and 57.9×, respectively (Fig. S11).

## DISCUSSION

**Fig 4:**
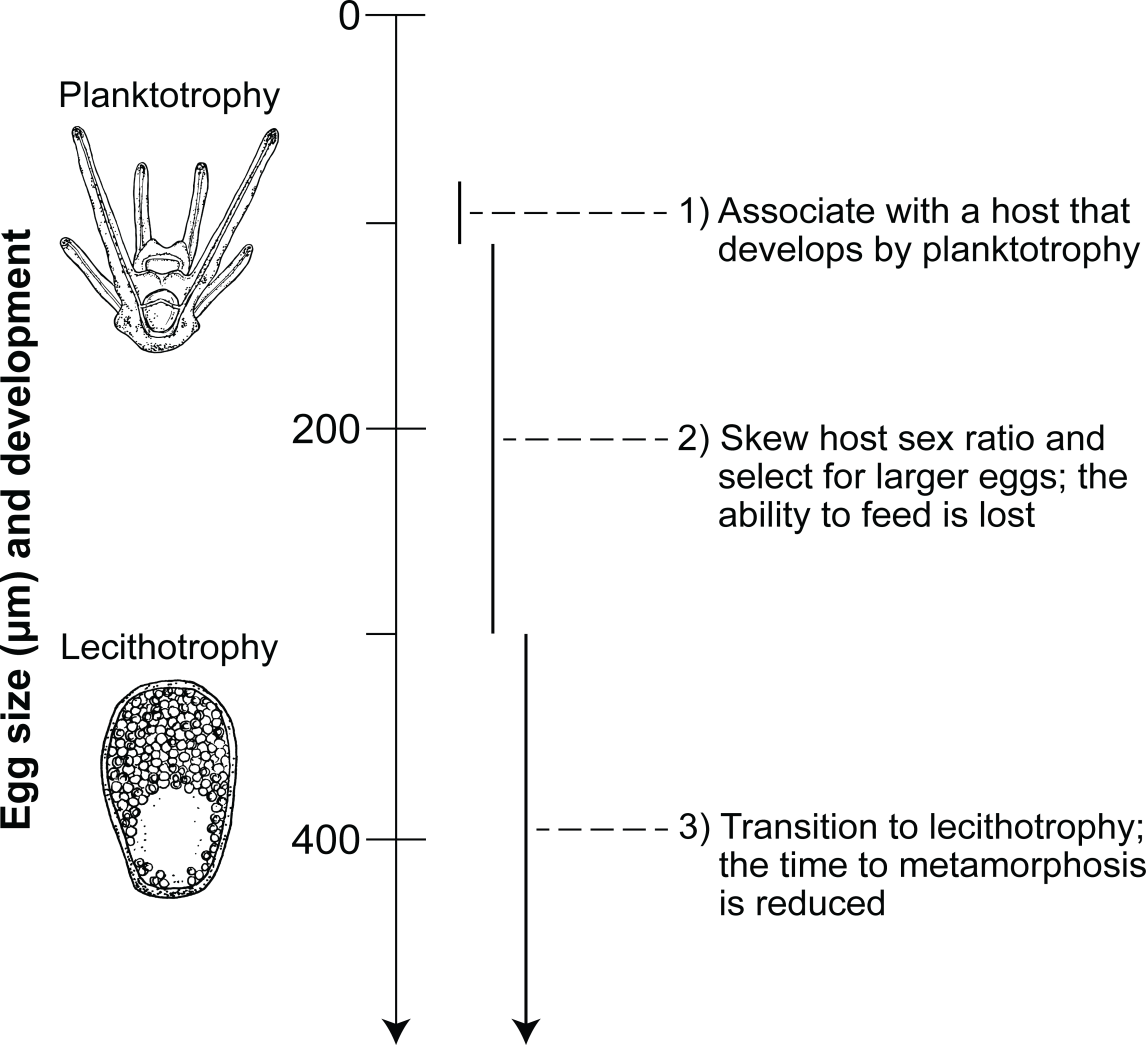
Theoretical schematic of how microbes manipulate developmental mode. A microbial manipulator associated with a planktotrophic (feeding) host would enhance its fitness by shifting the sex ratio toward female dominance. This favors larger eggs due to the increased chance of fertilization in a sperm-limited environment. Egg size in this female-dominant population would then stabilize between the fitness maxima of the host and the microbial manipulator. A substantial increase in maternal investment would relax the selective pressures that maintain the feeding structures and accelerate the time to metamorphosis. This, in turn, would lead to a derived—likely lecithotrophic (non-feeding)—developmental mode. Processes relating to the host reflect previous demonstrations of how developmental mode changes during these evolutionary transitions (6, 12, 49). Drawings by Alexandra Hahn.

Microbes manipulate host reproduction to enhance the number of microbial cells that are transmitted to the subsequent host generation (15, 16, 32). In this theoretical model, a microbe with the capability to manipulate host reproduction and engage in nutritional mutualism would associate with a marine invertebrate host that produces small eggs (~100 µm) and planktotrophic larvae (Fig. 4). The manipulator would then enhance its fitness by shifting the host’s sex ratio toward female dominance, which would favor larger eggs due to their increased chance of fertilization in a sperm-limited environment (Fig. 4) (43–45). The egg size of this female-dominant population would then stabilize within the fitness maxima for the host and the microbial manipulator (Fig. 4). A substantial increase in maternal provisioning (i.e., a larger egg) would relax the selective pressures that maintain the feeding structures and accelerate the time to metamorphosis. This, in turn, should lead to a derived—likely lecithotrophic—developmental mode (i.e., with an egg size >300 µm) (1, 12, 46, 47). Shortening this developmental window would limit the genetic exchange between populations, reduce the geographic range, and increase speciation and extinction rates (1, 2, 6, 24, 31, 48). Microbial manipulators represent a plausible evolutionary agent that, in turn, could drive an evolutionary transition between the major developmental modes among marine invertebrates.

Microbial manipulators are predicted to spread quickly and fixate within a population of free-spawning marine invertebrates (23). The theoretical models presented here suggest that individuals within this population would face a sperm-limited environment due to a microbe-induced distortion in the sex ratio, and then this population would undergo an evolutionary transition in developmental mode. This, in principle, may imply that the ancestral planktotroph would become extinct and be replaced by the derived lecithotroph. In the context of the best present example in the ocean: the ancestral *H. tuberculata* (a planktotroph) would have gone extinct and have been replaced by the derived *H. erythrogramma* (a lecithotroph) (20). Natural history data of the sea urchin *Heliocidaris* do not suggest a total species replacement (24, 50). Instead, this supports that the spread of these microbes within a population of the ancestral species is likely to be restricted to some degree (e.g., by suppression, resistance, or limited gene flow) (16, 51). The potential for the microbial manipulator to initially limit the reproductive success of the ancestral planktotroph and initiate some form of reproductive isolation within these overlapping populations (e.g., by cytoplasmic incompatibility) could allow for both host species to co-exist, undergo a rapid speciation event, and transition in developmental mode (16, 23, 52–55).

The loss of a microbial manipulator would theoretically allow for a balanced sex ratio to be re-established and for the host that recently experienced an evolutionary transition to either maintain this developmental mode or revert to the ancestral state. Phylogeny-based assessments of the developmental evolution in marine invertebrates support both possibilities and suggest that the option taken depends on the developmental constraints of that animal clade (5). The evolution of developmental modes is, for example, believed to be unidirectional in annelids and echinoderms and bidirectional in gastropods (3, 5, 11, 12). The loss of a microbial manipulator following a transition in developmental mode should result in annelids and echinoderms maintaining lecithotrophy, while gastropods should regain planktotrophy. An inconsistency in some life-history characters (i.e., sex ratio and sometimes egg size) may make it challenging to recognize former manipulations but may aid in the identification of a continued interaction following a microbe-induced evolutionary transition in developmental mode.

The broader applicability and robust testing of this theoretical model are partially dependent on identifying diverse hosts and reproductive manipulators. We used a combination of multiple life history characters (e.g., egg size and developmental mode), phylogeny, and geography to identify potential marine invertebrate genera that fit our theoretical framework for a microbe-induced transition in developmental evolution (9, 12, 31, 56–58). We suggest candidates from 17 genera representing annelids, echinoderms, and mollusks and from most major oceans (Table S4). These were predominantly echinoderms (82.4%; e.g., the sea star *Parvulastra* [56], the sea urchin *Heliocidaris* [20], and the brittle star *Macrophiothrix* [59]) and the majority were from Oceania (58.8%) (Table S4), an area of the world’s oceans with the richest developmental diversity (9, 56). Theory currently predicts that more than 80% of marine invertebrates could associate with microbial manipulators (23) and, thus, the available natural history data and well-resolved, species-level phylogenies for many marine invertebrate phyla likely limit our capacity to suggest additional candidates.

We describe a set of circumstances under which marine invertebrates could undergo an evolutionary transition between developmental modes, but the limitations of this theoretical model must also be considered. First, the biological data used to parameterize these models come primarily from sea urchins because of their widespread use in embryology, evolution, and ecology (23, 60, 61). Therefore, the implications of this model are more biased toward sea urchins than other groups of free-spawning marine invertebrates (e.g., annelids or gastropods) (1, 5, 11). Second, evolutionary transitions in developmental mode were modeled based on an egg size of 300 µm. This is, thus, an indirect assessment of the complexities involved in developmental evolution that is most strictly adhered to by sea urchins (6, 31). The specific conditions for a microbe-induced evolutionary transition in developmental mode would be expected to differ between major groups of marine invertebrates. Third, we only allowed for egg size to evolve in sperm-limiting conditions. What this model does not consider is how other gamete traits (e.g., affinity, chemotaxis, longevity, or quantity for sperm or jelly coat thickness for eggs) would also adapt to enhance fertilization in these conditions (43, 44).

Our theoretical model suggests that microbes can act as the evolutionary agent responsible for a transition in the reproductive strategy of marine invertebrates. We find it paramount to understand two fundamental questions. First, are there evolutionary transitions in developmental mode that are and are not the result of a microbial manipulator, and if so, how do they differ? Second, are the mechanisms of manipulation by terrestrial and marine microbes convergent or divergent? It appears within reason that evolutionary transitions in developmental mode may occur with and without reproductive manipulators because there are more documented evolutionary transitions in developmental mode among marine invertebrates than our suggested genera (5, 11, 12, 31, 56). We also find it within reason that terrestrial and marine microbes have converged on their mechanisms of manipulation because the suspected manipulator of the sea urchin *Heliocidaris*—*E*. *raffii*—is hypothesized to use a similar male-killing gene to the terrestrial *Spiroplasma* (20, 62). Thus, we anticipate that acknowledging microbial manipulators as a factor underlying the ecological and evolutionary processes governing marine invertebrate reproduction, development, and life history will shed light on age-old questions and conundrums in similar ways that they have for terrestrial life (1, 4, 6, 15–17, 33).

## MATERIALS AND METHODS

### Fertilization dynamics

We used a modified version of the Styan (61) polyspermy kinetic model to determine the fertilization dynamics of free-spawning marine invertebrates (23). Specifically, we converted egg (*E_T_;* eggs per μL) and sperm (*S_T_*; sperm per μL) concentrations to be explicitly determined by female and male density. We then determined the average number of sperm that may potentially fertilize an egg during a free-spawning event (*x*). This is a function of fertilization efficiency (*F_e_*), sperm concentration (*S_T_*; sperm per μL), egg concentration (*E_T_;* eggs per μL), the biomolecular collision constant (β_0_; mm^3^ per s), and the sperm half-life (τ; s):


(1) $$\begin{array}{c} x=F_{e}\frac{S_{T}}{E_{T}}\left(1-e^{-\beta_{o}E_{T}\tau }\right). \end{array}$$

Moreover, the biomolecular collision constant (β_0_) was determined by the cross-sectional area of the egg (σ; mm^2^) and sperm velocity (*ν*; mm per s):


(2) $$\begin{array}{c} \beta_{o}=\sigma \upsilon . \end{array}$$

The biomolecular collision constant and the time to elicit a block to polyspermy (*t_b_*; s) were then used to calculate the average number of extra sperm that may contact a fertilized egg, which then results in polyspermy (*b*):


(3) $$\begin{array}{c} b=F_{e}\frac{S_{T}}{E_{T}}\left(1-e^{-\beta_{o}E_{T}t_{b}}\right). \end{array}$$

By assuming a Poisson distribution for the arrival of sperm, the proportion of eggs that were fertilized by a single sperm and successfully developed was estimated by subtracting the probability of an egg being fertilized from the probability of an egg being fertilized by multiple sperm:


(4) $$\begin{array}{c} p\left(monospermic\;zygote\right)=1-e^{-x}-\left(1-e^{-x}-xe^{-x}\right)\left(1-e^{-b}\right). \end{array}$$

Zygote density (*N_z,t_*; zygotes per μL) was then estimated based on the probability of a monospermic zygote (equation 4) and total egg density (*E_T_*):


(5) $$\begin{array}{c} N_{z,t}=p\left(monospermic\;zygote\right)E_{T}. \end{array}$$

A summary of the variables and parameters is provided in Table S1.

### Population dynamics

We integrated the modified polyspermy kinetic model into a population model to simulate how a microbe that manipulates reproduction by feminization or male killing and can compensate for host fitness by supplementing host nutrition affects population size (23). This was a discrete-time population model with overlapping generations. Reproduction, development, and adult mortality are assumed to occur in that order because marine invertebrates tend to spawn seasonally (63).

Offspring survival based on egg size ($s_{\sigma }$) was determined using the Vance-Levitan survival function (33, 34, 60), where survival was determined by the survival function parameter (*B*) and egg size (σ):


(6) $$\begin{array}{c} s_{\sigma }=e^{-\frac{B}{\sigma }}. \end{array}$$

Specifically, *B* is the parameter determining the importance of egg size on survival, where larger values of *B* equate to egg size being more important to offspring survival (Fig. S1A).

Adult mortality (*M_a_*) was assumed to be density-dependent and based on the maximum adult mortality rate (*m_a_*), carrying capacity (*k*; individuals per m^2^), the total density of the population at time *t* (*N_t_*; individuals per m^2^), and the degree to which density influences mortality (*d;* i.e*.*, the shape parameter). Both the current adult density and density of surviving zygotes that settled were assumed to influence density dependence, where *N^′^_z,t_* is the density of zygotes (zygotes per μL) that survived after a microbe-induced male killing and ψ (μL per m^2^) is the settlement constant that describes the number of surviving zygotes that transitioned from larvae to juveniles:


(7) $$\begin{array}{c} M_{a}=\left[\frac{m_{a}}{1+e^{-d\left(N_{t}+N_{z,t}^{\prime }s_{\sigma }\psi -\frac{k}{2}\right)}}\right]. \end{array}$$

The density of females in the next generation (*N_f,t+1_*; individuals per m^2^) was then determined by the density of surviving female zygotes (*N_z,f,t_*; zygotes per μL) that settled plus the density of surviving female adults at generation *t* (*N_f,t_*; individuals per m^2^):


(8) $$\begin{array}{c} N_{f,t+1}=\left(N_{z,f,t}L_{\sigma }\psi +N_{f,t}\right)\;\left(1-M_{a}\right). \end{array}$$

For male killing scenarios, we assumed that females produced an equal number of daughters and sons and, therefore, the density of female zygotes (*N_z,f,t_*) is calculated by dividing the density of zygotes (*N_z,t_*) by two. The density of males in the next generation (*N_m,t+1_*; individuals per m^2^) has the same structure as equation 8. The density of male zygotes (*N_z,i,m,t_*; zygotes per μL) is influenced by mortality due to male killing (*m_k_*):


(9) $$\begin{array}{c} N_{z,m,t}=\frac{N_{z,t}\left(1-m_{k}\right)}{2}. \end{array}$$

For feminization scenarios, the sex ratio of zygotes was biased by feminization rate *r* such that the density of female zygotes (*N_z,f,t_*) was:


(10) $$\begin{array}{c} N_{z,f,t}=rN_{z,t} \end{array}$$

and the density of male zygotes (*N_z,m,t_*) was:


(11) $$\begin{array}{c} N_{z,m,t}=\left(1-r\right)N_{z,t}. \end{array}$$

A summary of the variables and parameters is provided in Table S2.

### Grid-based invasion model

We performed a deterministic grid-based invasion analysis to simulate the population dynamics for free-spawning marine invertebrates along a continuum of egg sizes (*σ*)—from 50 µm to 1,500 µm at increments of 10 µm (9)—until an equilibrium was reached (i.e., a change in population densities was equivalent to zero) for a population with or without a microbe that can manipulate reproduction by feminization or male killing and/or enhanced growth. This approach was chosen instead of an adaptive dynamics approach because it was not feasible to find an analytical solution for density given the complex fertilization equations. We then calculated the number of surviving fertilized eggs (i.e., fitness) of an individual that produced the resident egg size—the current position on the grid—and compared this to the fitness of a mutant individual that produced an egg size that was either 10 µm smaller or larger at each point on the grid. Fitness of a given egg size (*W_σ_*) was a function of the probability of fertilization success (*P_f_,_σ_*), egg density (*E_σ_*; egg per µL), and survival (*L_σ_*) (Fig. 1A):


(12) $$\begin{array}{c} W_{\sigma }=P_{f,\sigma }E_{\sigma }s_{\sigma }. \end{array}$$

The probability of fertilization success was calculated using the resident egg number because we assume that the mutant is rare (34), but egg size was varied based on whether the calculation was for a resident, smaller mutant, or larger mutant. Egg volume is inversely proportional to fecundity (35) and, thus, we calculated the density of eggs produced using a constant for planktotrophs (i.e., 0.112) divided by egg volume (*σ*) and multiplying that by the enhanced growth rate factor (*g*). We performed this invasion grid analysis to solve for the optimal egg size across male killing rates (0–0.99 at increments of 0.03), feminization (0.5–0.995 at increments of 0.015), enhanced growth rates (0–3 at increments of 0.1), and *B* values (100–3,000 at increments of 100).

We then calculated fitness for the host across all model parameters to determine this fitness landscape. Host fitness was calculated using equation 12 assuming the evolutionary stable egg size. Fitness values were then compared between a host that was (*W_i_,σ*) and was not (*W_u_,σ*) associated with a microbial manipulator to assess how an evolutionary shift in egg size changed the fitness landscape:


(13) $$\begin{array}{c} W_{r,H,\sigma }=\frac{W_{i,\sigma }}{W_{u,\sigma }}. \end{array}$$

All simulations were run in R (v. 3.5.0).

### Quantitative genetic population model

We developed a deterministic quantitative genetic population model to numerically simulate the evolution of egg size (*σ*) in a population of free-spawning marine invertebrates. To approximate a continuous normal distribution of female egg size, we created bins of egg volume (*σ*) that were 0.2 µm in size, which resulted in 14,950 bins across the range of possible egg sizes in the model (10–3,000 µm). We initialized populations with a mean of 180 µm, a standard deviation of 9 µm, and a range of ±10 standard deviations from the mean. To generate starting numbers of individuals at each egg size bin, we calculated the relative Gaussian probability density function multiplied by the total population size (1 individual per m^2^). Males in the population were assumed to not vary in any reproductive trait. Fitness for each egg size bin was a product of fertilization success (equation 5), larval survival based on egg size (equation 6), and the settlement constant ψ (μL per m^2^).

Microbial abundance (i.e., titer) and the functional capacity of that microbial population scale allometrically with offspring size (40–42). Microbial manipulators can exploit this relationship because their fitness depends on the number of cells that are transmitted between generations by increasing offspring size and fitness (38). We accounted for the influence of microbial manipulators on the relationship between offspring size and fitness by establishing the *V* parameter. The V parameter represents the egg size where the B parameter is influenced halfway to the maximal possible influence. This relationship was modeled using a Michaelis-Menten equation and, thus, can be more precisely termed the effective *B* parameter (*B_v_*) (Fig. S5A) (64):


(14) $$\begin{array}{c} B_{v}=\frac{3,000\sigma }{V+\sigma }. \end{array}$$

The influence of microbial abundance on larval survival was then accounted for by adding *B_v_* and *B* to equation 6 (Fig. S5B):


(15) $$\begin{array}{c} L_{\sigma }=e^{-\frac{\left(B+B_{v}\right)}{\sigma }}. \end{array}$$

We then calculated the fitness (i.e., the number of surviving offspring) for each egg size bin and the egg size distribution of offspring using standard quantitative genetics (65). Specifically, the selection differential on egg size (*S_σ_*) was calculated as a function of mean fitness (*w*) and covariation of fitness and egg size (*σ*):


(16) $$\begin{array}{c} S_{\sigma }=\frac{1}{\overset{-}{w}}cov\left(w,\sigma \right) \end{array}$$

The response to selection (*R*) was calculated as the heritability (*h^2^*) multiplied by the selection differential (i.e., the quantitative genetic breeder’s equation):


(17) $$\begin{array}{c} R=h^{2}s_{\sigma } \end{array}$$

We assumed that heritability was constant at 0.05. Preliminary analyses of varying heritability influenced the quickness of egg size evolution and not stable egg size outcomes. We then generated the female offspring distribution with a mean of the previous generation plus *R*, a standard deviation of 9 µm, and a total density equal to the density of surviving female offspring produced accounting for either feminization or male killing and enhanced growth.

For male killing scenarios, we assumed half of the surviving offspring were males and the other half females (equation 9). The surviving males experienced a male-killing mortality rate (*m_k_*). For feminizing scenarios, where *r* represents the feminization rate: *r* proportion of surviving offspring were female and 1-*r* surviving offspring were male (equation 10). The density of each egg size bin for females in the next generation was added to surviving individuals from the previous generation. The density of males in the next generation was calculated by adding the density of surviving male offspring to the density of survival males from the previous generation (equation 11). For simplicity, we assumed a constant adult mortality rate (*m_a_*) of 0.9. Initial simulations varying this number did not qualitatively affect the results. If the total population size after mortality exceeded the set carrying capacity (*k*), then the population size was adjusted to *k*. Simulations were run for 4,000 generations, as mean egg size stabilized well before then across a wide range of parameters. Fertilization parameters were the same as the invasion grid model (Table S1).

A summary of parameter values specific to the quantitative genetic model is given in Table S3. All simulations were run using Julia (v. 1.9.2).

### Identification of candidate manipulations

We used the literature on marine invertebrate egg size (9, 31, 58), developmental mode (9, 31, 58), phylogeny (56, 57), and geography (via World Register of Marine Species) to suggest candidate transitions in developmental mode that may have been the result of a microbial manipulation. We define a candidate microbial manipulation of developmental mode as the ancestral host species having an egg size of ~100 µm (i.e., a normal size for obligate planktotrophy [6, 31] and within the first maxima in relative fitness [Fig. 1 to 3]) and the derived species having an egg size between ~300 and ~500 µm (i.e., the smallest egg size for lecithotrophy and within the second maxima in relative fitness [Fig. 1 to 3]). Moreover, sister species also had to exhibit an evolutionary transition in developmental mode (from planktotrophy to lecithotrophy) and inhabit a similar geographical region (see Table S4). Additional suggested taxa of interest were genera that nearly met these qualifications (see Table S4).

## Data Availability

All computer code and data to support the analyses and conclusions of this study are available online on the Dryad Digital Repository: https://doi.org/10.5061/dryad.0p2ngf27f.

## SUPPLEMENTAL MATERIAL

Supplemental material mbio.03655-24-s0001.pdf Supplemental tables and figures.

ASM does not own the copyrights to Supplemental Material that may be linked to, or accessed through, an article. The authors have granted ASM a non-exclusive, world-wide license to publish the Supplemental Material files. Please contact the corresponding author directly for reuse.
