# Supplementary material for: Microbes as manipulators of egg size and developmental evolution
Source: mBio. 2025 Apr 17;16(5):e03655-24. doi: 10.1128/mbio.03655-24 (PMC12077187; doi:10.1128/mbio.03655-24)
Supplement: Supplemental material — Supplemental tables and figures. [file mbio.03655-24-s0001.pdf]

Supplemental Information for:

Microbes as manipulators of egg size and developmental evolution

Matthew C. Kustra[1,2*] and Tyler J. Carrier[3,4*]

[1] Department of Ecology and Evolutionary Biology, University of California, Santa Cruz, USA

[2] Department of Integrative Biology, University of California, Berkeley, USA

[3] GEOMAR Helmholtz Centre for Ocean Research, Kiel, Germany

[4] Zoological Institute, Kiel University, Kiel, Germany

[*] Corresponding authors: matthewckustra@berkeley.edu; tcarrier@geomar.de

This file includes:

- Supplementary Tables (S1 to S4)
- Supplementary Figures (S1 to S11)

SUPPLEMENTARY TABLES

Table S1. Definitions and parameters values for fertilization dynamics adapted from Styan (60).

Symbols	Definition	Values
σ	Cross-sectional area of egg (mm^2)	0.04-1.5 (0.01)[†]
v	Sperm swimming speed ($mm\ s^{-1}$)	0.140 (66)
E	Egg density per unit female density (egg mL^{-1} per indiv. m^{-2})	0.112[‡]/σ
S	Sperm density per unit male density (sperm mL^{-1} per indiv m^{-2})	700 (54)
F_e	Fertilization efficiency	0.094[*]
τ	Sperm half-life (s)	5,400 (67)
t_b	Time for polyspermy block (s)	1 (68)
β_0	Biomolecular collision constant ($mm^3\ s^{-1}$)	σv
x	Average number of potential fertilizing sperm	15
b	Average number of extra fertilizing sperm that contacts an egg	Equation 3

Note: [†] denotes that these egg diameter (mm) values were converted to cross-sectional area of egg (mm^2) during the model. [‡] This value was calculated by multiplying egg density and egg volume to keep the overall fecundity for different egg sizes the same. [*] denotes unpublished data provided by Maria Byrne (University of Sydney, Australia).

Table S2. Definitions and values of parameters for non-stage-based population dynamics model.

Symbols	Definition	Values
Parameters		
k	Adult carrying capacity (indiv. m^{-2})	100
ψ	Settlement constant (mL m^{-2})	1
d	Mortality rate of change	0.1
m_a	Max adult mortality	0.99
B	Importance of egg size	300 – 3,000 (300)
g	Enhanced growth (fecundity enhancement, %)	0-3 (0.1)
r	Feminization rate	0.5 – 0.99 (0.06)
m_k	Male killing rate	0 – 0.99 (0.03)
Functions		
$L\sigma$	Larval survival	Equation 6
M_a	Adult mortality	Equation 7
Variables		
N_t	Density of all adult indiv. at time t	0.2
$N_{f,t}$	Density of adult females at time t	0.1
$N_{m,t}$	Density of adult males at time t	0.1
$N_{z,t}$	Density of initial zygotes at time t	N/A
$N^*_{z,t}$	Density of zygotes at time t after male killing	N/A
$N_{z,f,t}$	Density of new females at time t	N/A
$N_{z,m,t}$	Density of new males at time t	N/A

Note: Capital letters are functions or variables, and initial values are given when available. Zygote density units are zygotes per μL and adult density units are individuals per m^2. Numbers in parenthesis indicate step size in sensitivity analyses.

Table S3. Definitions and values of parameters for quantitative genetic population model.

Symbols	Definition	Values
Parameters		
k	Adult carrying capacity (indiv. m^{-2})	2.6
ψ	Settlement constant (mL m^{-2})	1,000
m_a	Adult mortality	0.9
h^2	Heritability	0.05
B	Importance of egg size	250 – 750 (10)
g	Enhanced growth (fecundity enhancement, %)	0 – 2 (0.5)
m_k	Male killing rate	0 – 0.98 (0.02)
r	Feminization rate	0.5 – 0.99 (0.01)
V	Rate parameter determining how egg size influences *B*.	250 – 750 (10)
Functions		
B_v	Effective *B* parameter modifier	Equation 14
$L\sigma$	Larval survival	Equation 15
$S\sigma$	Selection differential	Equation 16
R	Response to selection	Equation 17

Note: Capital letters are functions or variables, and initial values are given when available. Zygote density units are zygotes per μL and adult density units are individuals per m^2. Numbers in parenthesis indicate step size in sensitivity analyses.

Table S4. Candidate marine invertebrate genera of a microbe-mediated evolutionary transition in developmental mode.

Candidate taxa

Phylum	Class	Genus	Region	References
Echinodermata	Asteroidea	*Astropecten*	NW Pacific	(69)
		Cryptasterina	Oceania	(70)
		Meridiastra	Oceania	(55)
		Parvulastra	Oceania	(55, 71)
	Echinoidea	*Heliocidaris*	Oceania	(20, 49)
	Holothuroidea	*Holothuria*	Caribbean	(72, 73)
	Ophiuroidea	*Macrophiothrix*	Oceania	(56, 58)
		Ophiocoma	Oceania	(74)
		Ophiomastix	Oceania	(74)
		Ophionereis	Oceania	(75)
Molluca	Gastropoda	*Crepidula*	Atlantic<br>Pacific	(11, 76)

Additional taxa-of-interest

Phylum	Class	Genus	Region	References
Annelida	Polychaeta	*Diopatra*	N Atlantic	(9)
		Platynereis	Global	(77)
Echinodermata	Asteroidea	*Aquilonastra*	Oceania	(55)
		Asterina	N Atlantic	(55, 71)
	Echinoidea	*Clypeaster*	Caribbean	(78)
		Phyllacanthus	Oceania	(79, 80)

SUPPLEMENTARY FIGURES

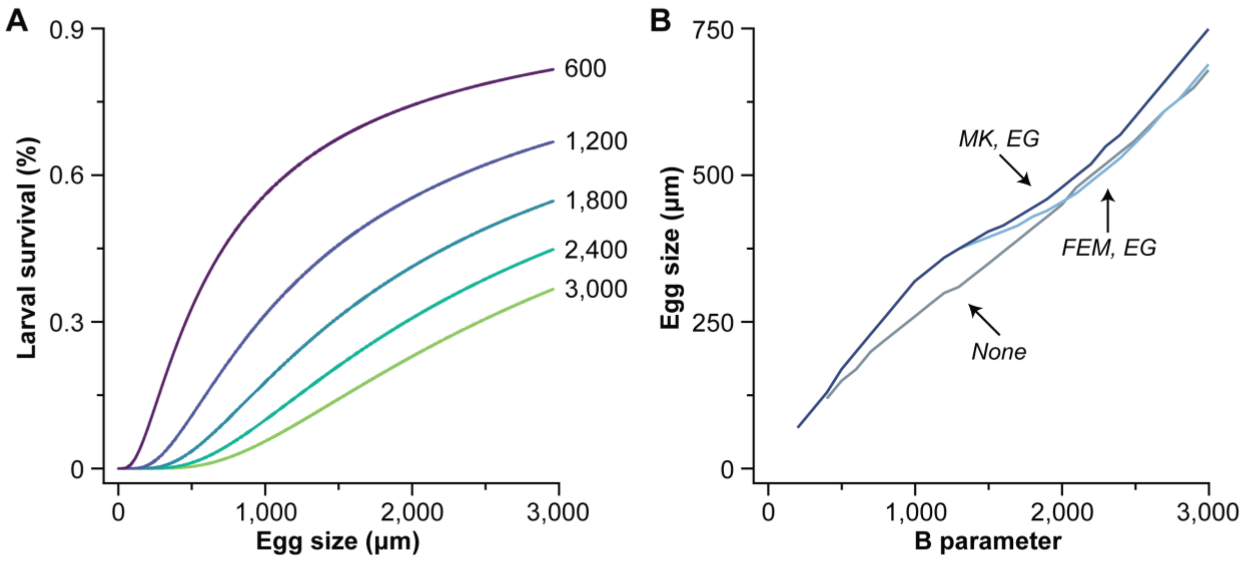

Fig S1. Relative importance of the *B* parameter to egg size and larval survival. (A) The relationship between egg size and larval survival at *B* values from 600 to 3,000 using Equation 6. (B) Stable egg size (μm in diameter) across *B* parameter (*i.e.*, the offspring survival function) values for populations without a manipulator (gray) compared to those with a feminizing (light blue) or male killing (dark blue) manipulator that also enhances growth.

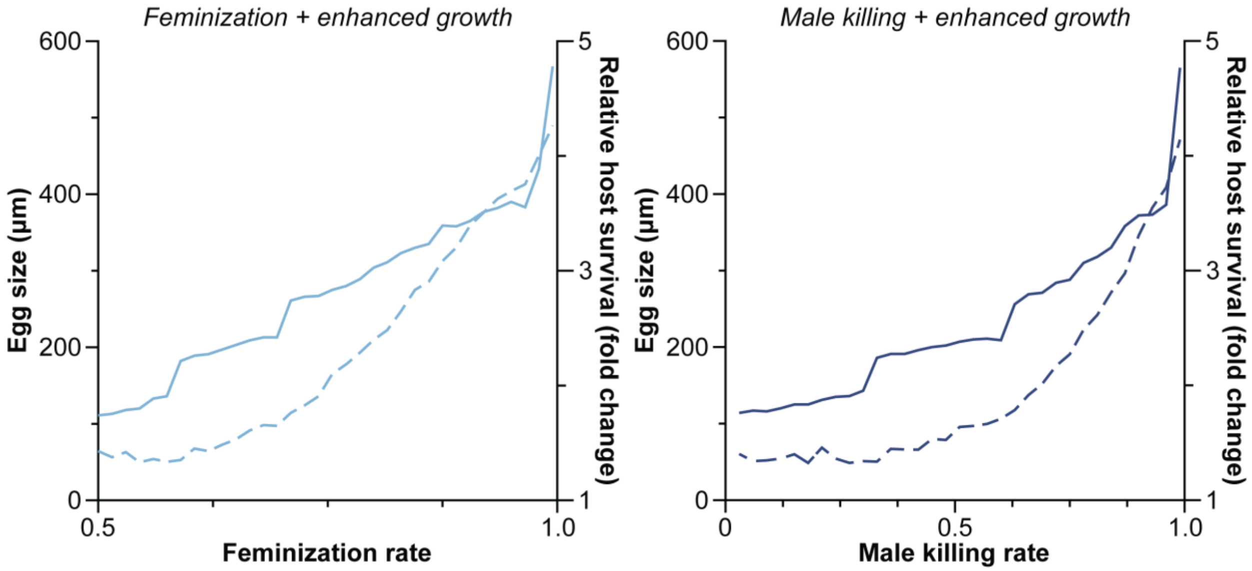

Fig S2. Egg size and magnitude of the second peak in relative survival increases with manipulation rate. The evolutionary shift in stable egg sizes due to a microbial manipulator results in two fitness peaks, one representing planktotrophy and another for lecithotrophy (see, Fig 1C). The magnitude in relative fitness (*i.e.*, manipulated compared to un-manipulated; dashed line) for this 'lecithotrophy' peak directly relates to manipulation rate as well as the egg size (solid line) at which that peak occurs. As such, the peak in relative survival and the egg size of that peak increases with manipulation rate. This was consistent for a manipulator with the capacity for feminization (left) or male killing (right) and can compensate host fitness by supplementing host nutrition.

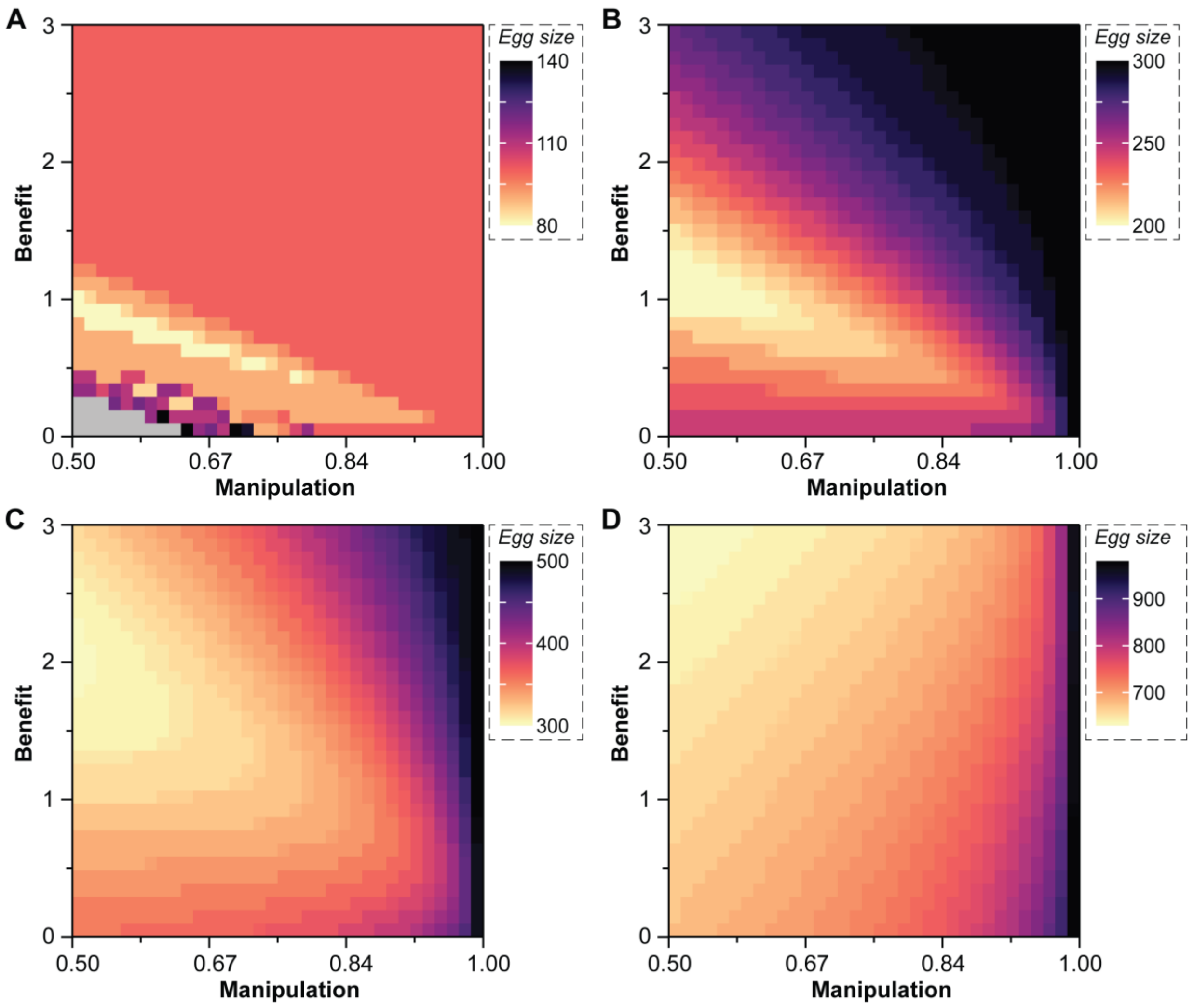

Fig S3. Influence of *B* parameter, feminization, and enhanced growth on egg size evolution. Evolutionary stable egg size (μm in diameter) across feminization (0.50 to 0.99 at increments of 0.03) and enhanced growth rates (0 to 3.0 at increments of 0.1) when the *B* parameter equals 300 (A), 900 (B), 1,500 (C), or 3,000 (D). Evolutionary stable egg sizes were determined using the invasion grid analysis model. Grey shading indicates parameter combinations that resulted in unviable populations.

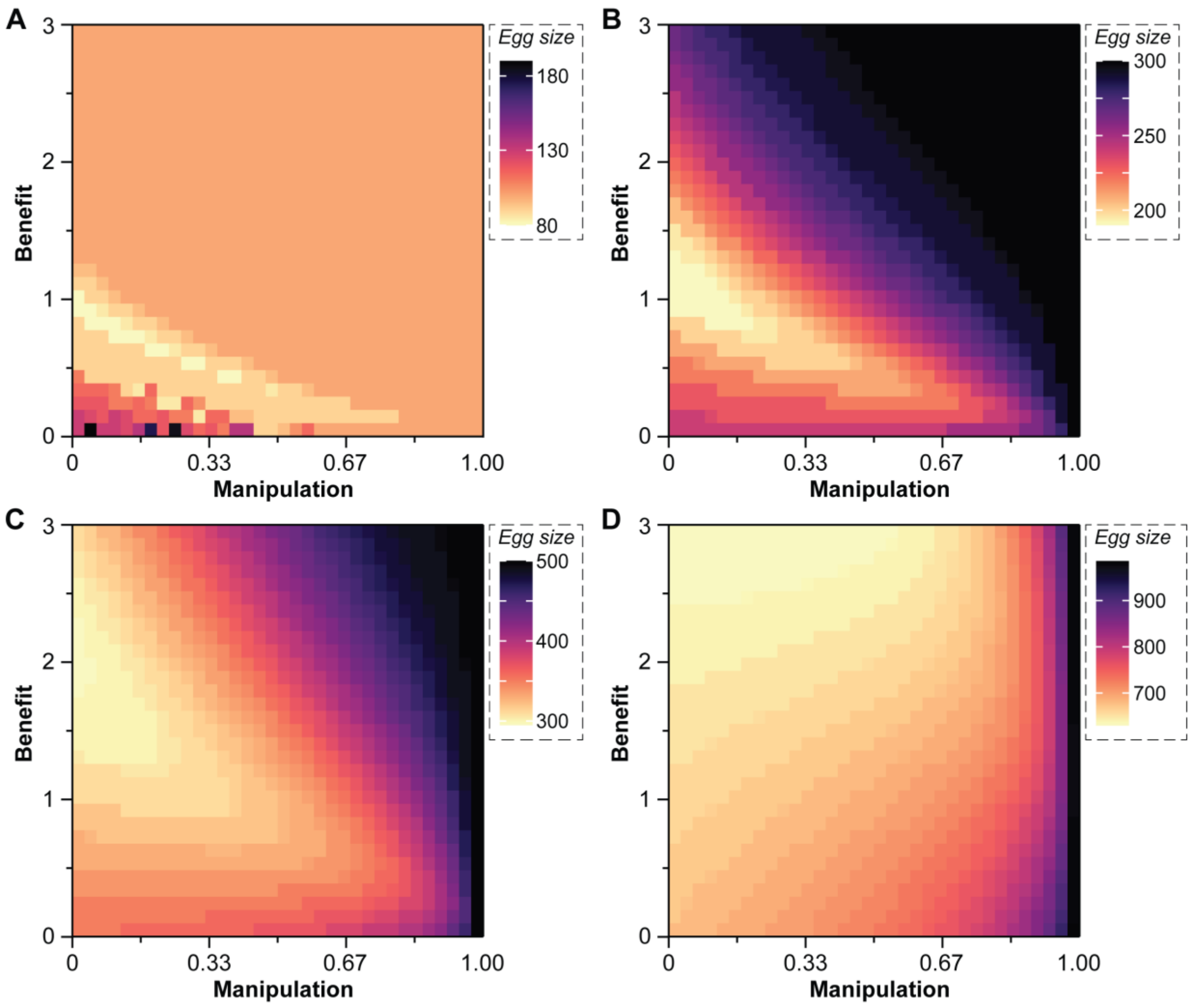

Fig S4. Influence of *B* parameter, male killing, and enhanced growth on egg size evolution. Evolutionary stable egg size (μm in diameter) across male killing (0 to 0.99 at increments of 0.03) and enhanced growth rates (0 to 3.0 at increments of 0.1) when the *B* parameter equals 300 (A), 900 (B), 1,500 (C), or 3,000 (D). Evolutionary stable egg sizes were determined using the invasion grid analysis model. Grey shading indicates parameter combinations that resulted in unviable populations.

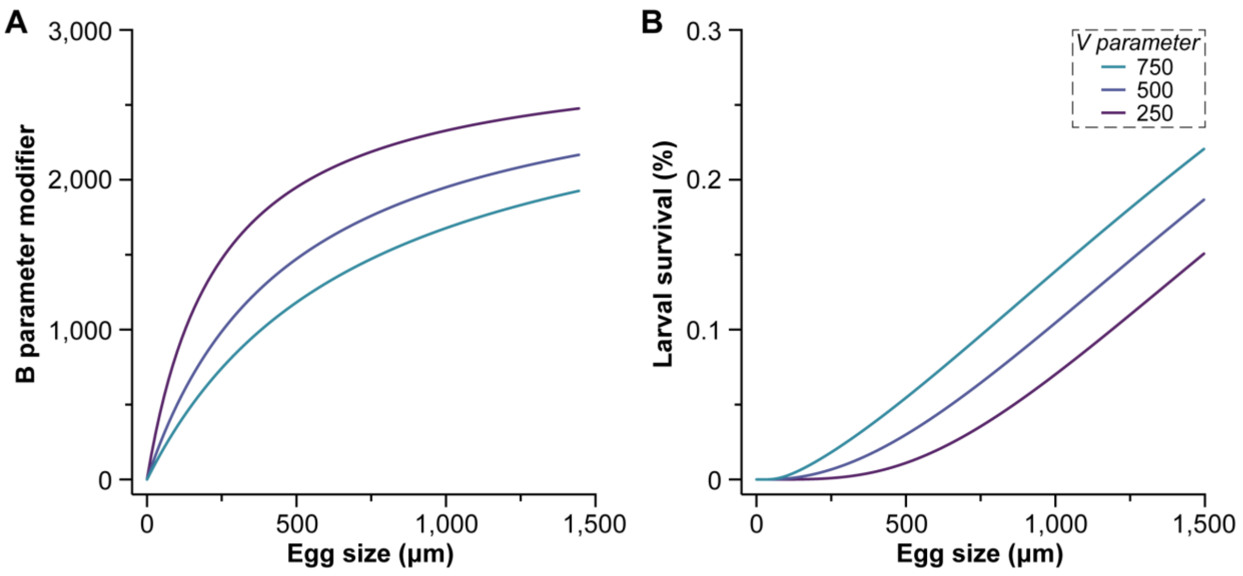

Fig S5. ***V*** **parameter determines how egg size modifies the** ***B*** **parameter and the relationship between egg size and larval survival.** (A) The relationship between egg size and how the *B* parameter is modified at different *V* values using Equation 14. (B) The relationship between egg size and larval survival at different *V* values when *B* = 250 (Equation 15).

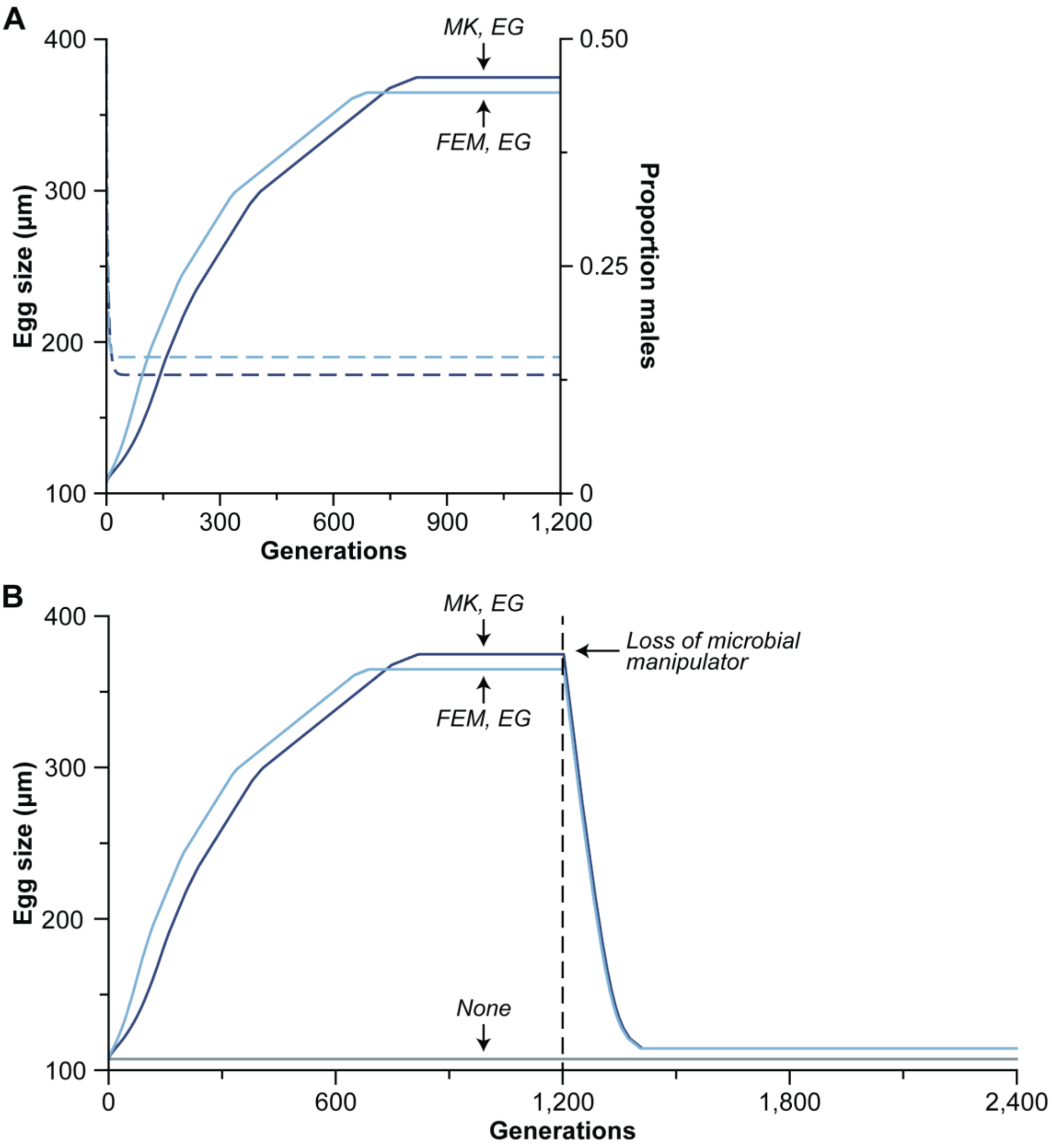

Fig S6. Speed and reversal of a microbe-induced evolutionary transition in developmental mode. (A) If a free-spawning marine invertebrate associates with a microbe that has the capacity for male killing (MK; dark blue; solid line) or feminization (FEM; light blue; solid line) and for enhanced growth (EG; *i.e.*, a nutritional symbiosis), then egg size (µm in diameter) increases enough that the host can undergo an evolutionary transition in developmental mode. This change in egg size and development is preceded by a shift in sex ratio (dashed lines) that is caused by the microbial manipulator. (B) Removal of a microbe that manipulates by feminization or male killing and enhanced growth from a stable population following an evolutionary transition in developmental mode. Egg size following removal of the microbial manipulator nearly results in a return to a population that never associated with a microbial manipulator (gray). Parameter values are the same as Fig 2 before loss of microbial manipulator.

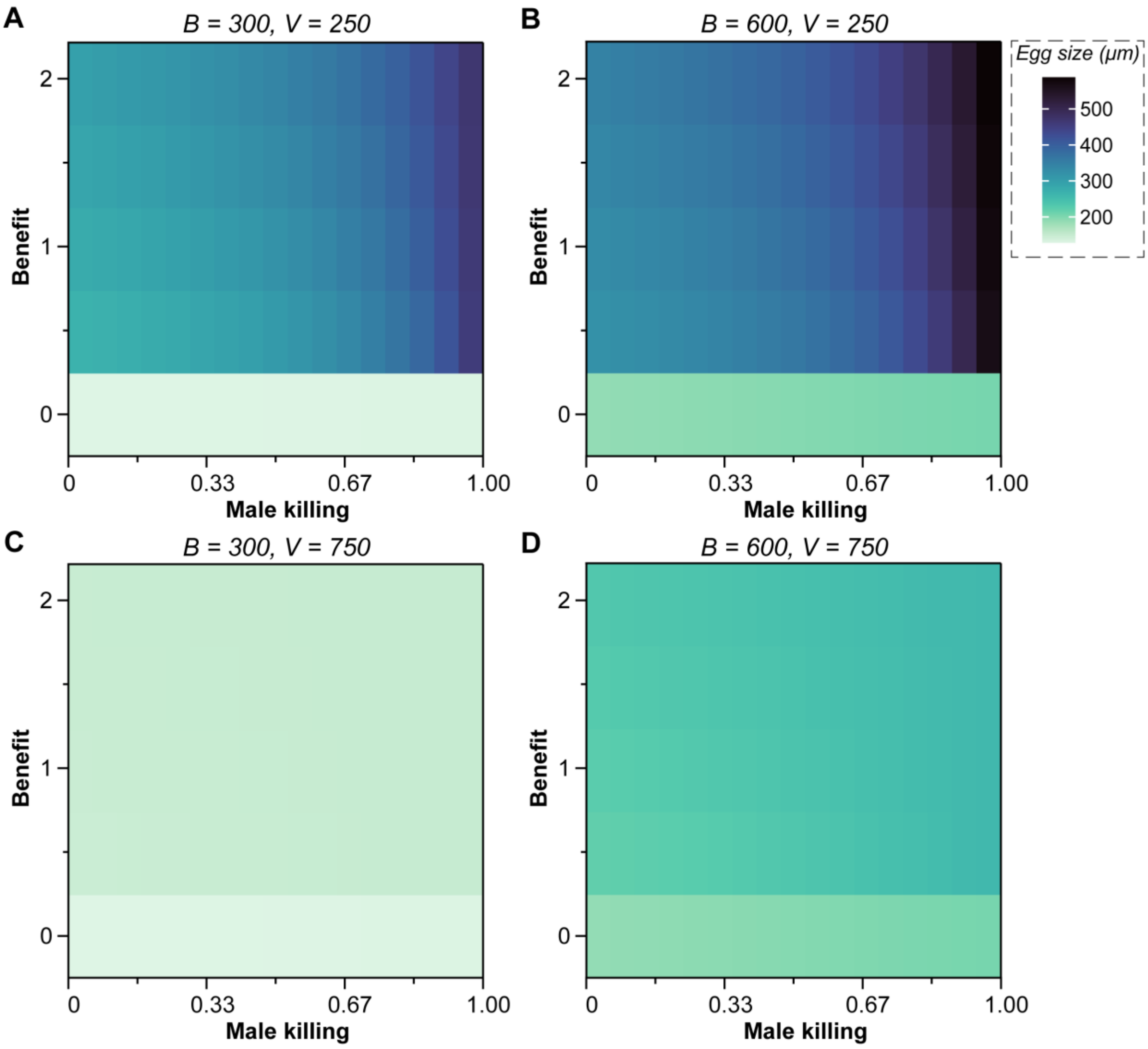

Fig S7. Presence, not magnitude, of enhanced growth influences stable egg size. Heat map of stable egg sizes at different male killing rates and enhanced growth factors across different combinations of *B* and *V* values.

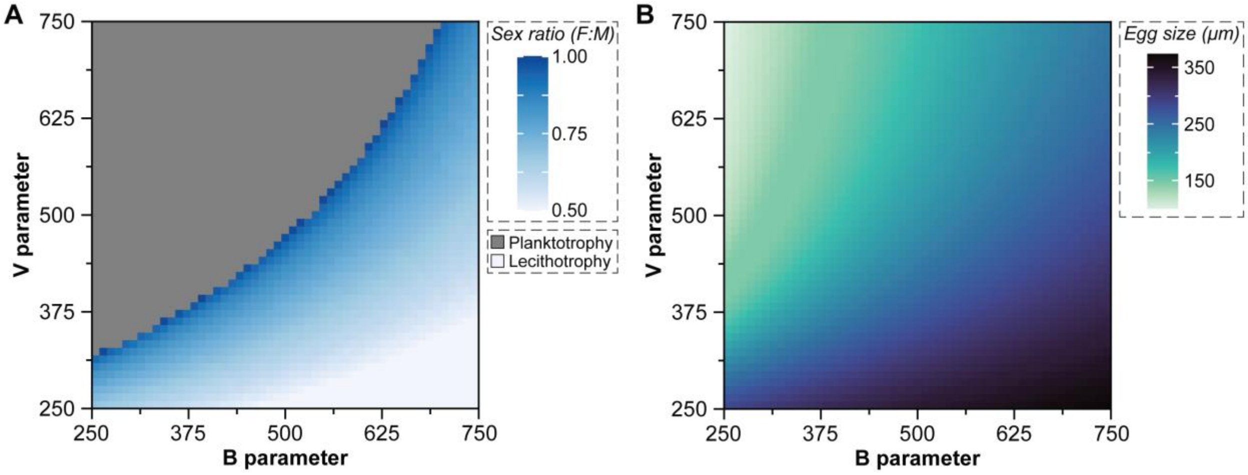

Fig S8. Feminization and enhanced growth results in similar patterns as male killing and enhanced growth. The conditions for a microbe-mediate evolutionary transition in developmental mode depends on the ecological conditions of the host (*i.e.*, *B* parameter) as well as the manipulating capability of the microbe (*i.e.*, *V* parameter). These factors create a gradient in the sex ratio (A) needed to induce an evolutionary transition in developmental mode (*i.e.*, at 300 μm) as well as a gradient in the stable egg sizes that follow this transition (B), with the mean egg size for each combination presented here. No evolutionary transition may also be observed when ecology heavily favors planktotrophy and the manipulating capability of the microbe is weak (gray, top left of A). When ecology favors larger egg sizes, only enhanced growth is needed for an evolutionary transition to occur and no manipulation in sex ratio (light blue, bottom right of A). The microbe here represents feminization and enhanced growth, but a nearly identical pattern is observed for male killing and enhanced growth (Fig 3).

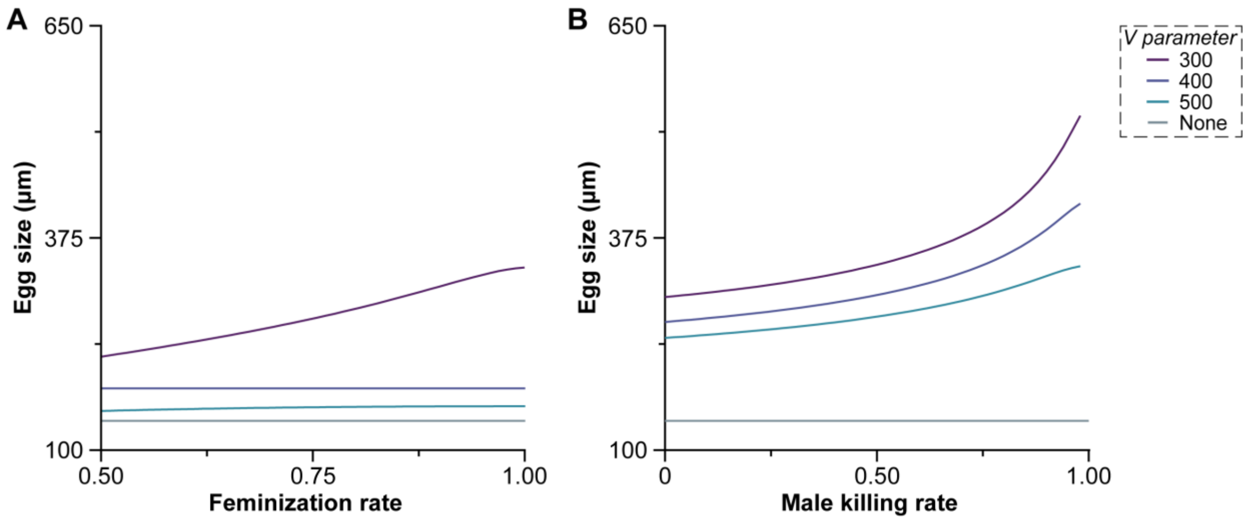

Fig S9. Egg size (µm in diameter) increases with feminization (left) and male killing (right) rates. Egg size increases at a given manipulation rate as *V* decreases. *B* parameter for this graph is set at 250. Grey line indicates evolutionary stable egg size for when there is no microbial association.

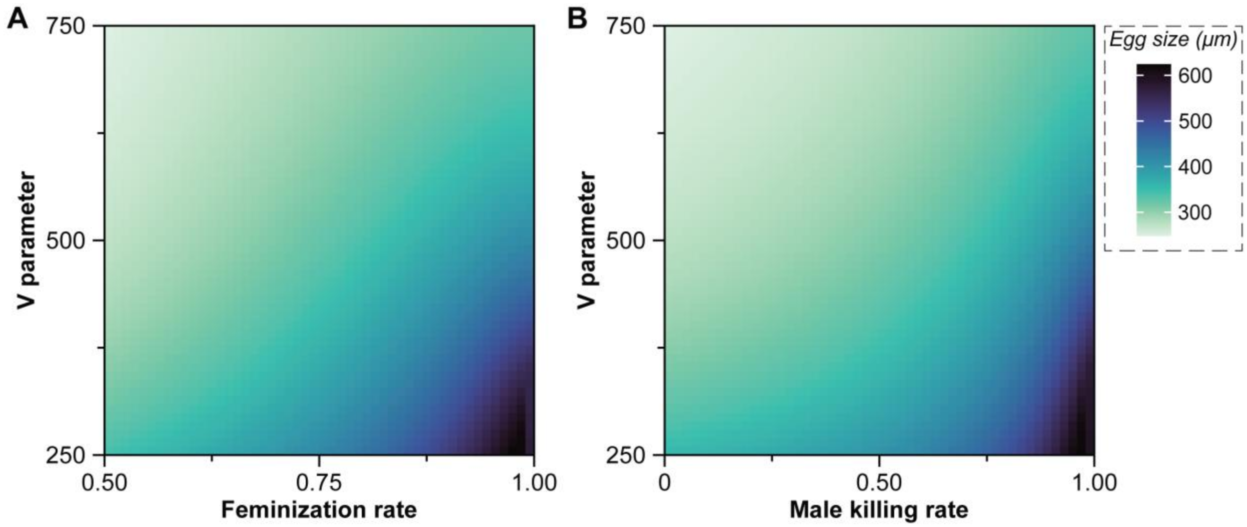

Fig S10. Stable maximum egg size increases with microbial manipulation and decreases with *V* parameter. This pattern is similar for a microbe that manipulates host reproduction by feminization (A, left) or male killing (B, right).

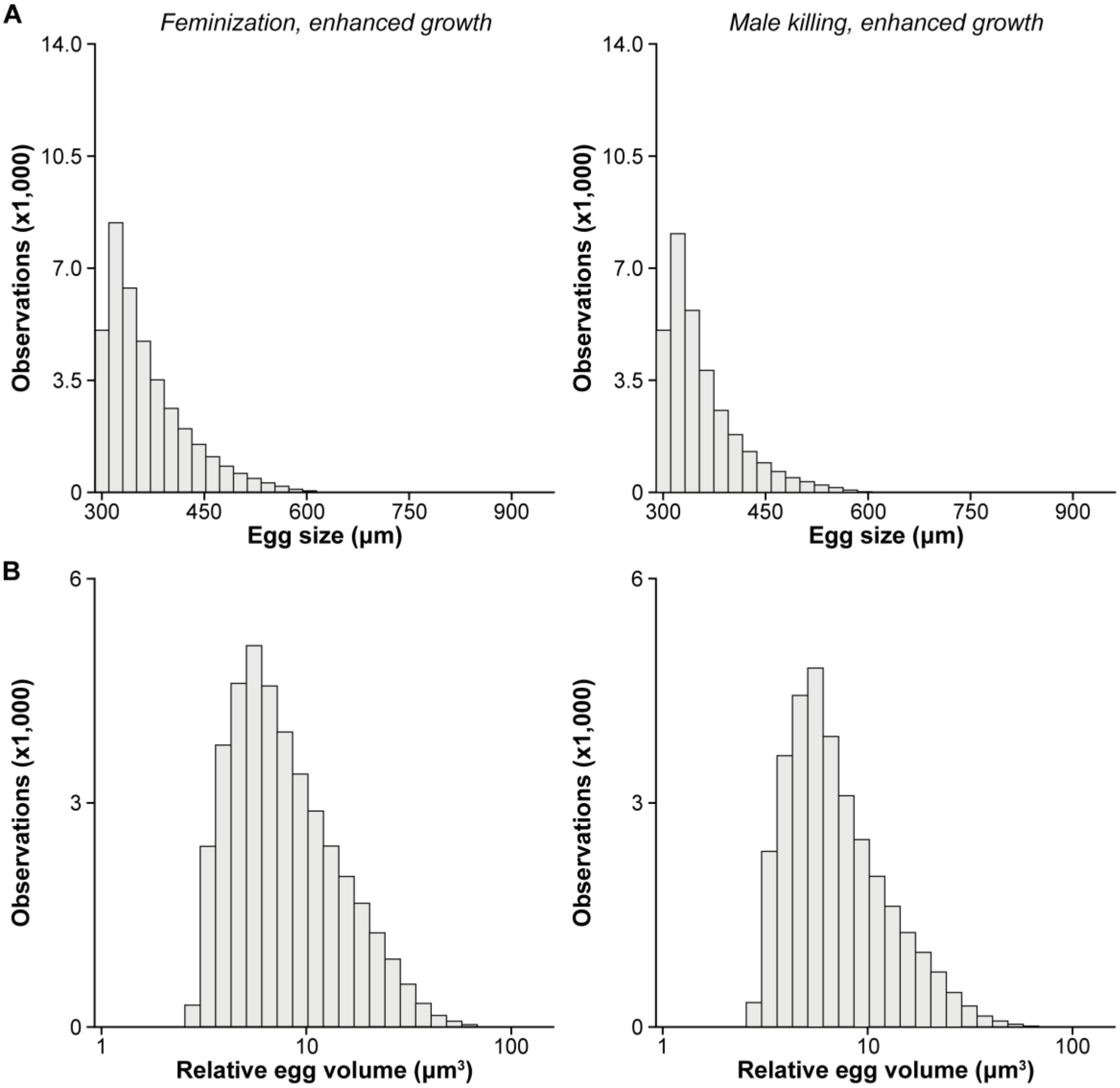

Fig S11. Stable egg sizes following a microbe-mediate evolutionary transition in developmental mode. Distribution of stable egg sizes (μm; A, top) and relative egg volume (μm^3; B, bottom) following an evolutionary transition in developmental mode that was induced by a microbe that manipulates host reproduction by feminization (left) or male killing (right) and can compensate host fitness by supplementing host nutrition. These distributions represent the full range of stable egg sizes, as opposed to Fig 3 that present the mean value for a given combination of the *B* and *V* parameters.

REFERENCES

9. Marshall D, Krug P, Kupriyanova E, Byrne M, Emlet R. 2012. The biogeography of marine invertebrate life histories Annual Review of Ecology, Evolution, and Systematics 43:97-114.

11. Collin R. 2003. Worldwide patterns in mode of development in calyptraeid gastropods. Marine Ecology Progress Series 247:103-122.

19. Carrier T, Leigh B, Deaker D, Devens H, Wray G, Bordenstein S, Byrne M, Reitzel A. 2021. Microbiome reduction and endosymbiont gain from a switch in sea urchin life-history. Proceedings of the National Academy of Sciences 118:e2022023118.

48. Hart M, Abt C, Emlet R. 2011. Molecular phylogeny of echinometrid sea urchins: more species of *Heliocidaris* with derived modes of reproduction. Invertebrate Biology 130:175-185.

53. Evans J, Marshall D. 2005. Male-by-female interactions influence fertilization success and mediate the benefits of polyandry in the sea urchin *Heliocidaris erythrogramma*. Evolution 59:106-112.

54. Byrne M. 2006. Life history diversity and evolution in the Asterinidae. Integrative and Comparative Biology 46:243-254.

55. Hart M, Podolsky R. 2005. Mitochondrial DNA phylogeny and rates of larval evolution in *Macrophiothrix* brittlestars. Molecular Phylogenetics and Evolution 34:438-447.

57. Allen J, Podolsky R. 2007. Uncommon diversity in developmental mode and larval form in the genus *Macrophiothrix* (Echinodermata: Ophiuroidea). Marine Biology 151:85-97.

59. Styan C. 1998. Polyspermy, egg size, and the fertilization kinetics of free-spawning marine invertebrates. The American Naturalist 152:290-297.

66. Foo S, Deaker D, Byrne M. 2018. Cherchez la femme – impact of ocean acidification on the egg jelly coat and attractants for sperm. Journal of Experimental Biology 221:13.

67. Binet M, Doyle C. 2013. Effect of near-future seawater temperature rises on sea urchin sperm longevity. Marine and Freshwater Research 64:1-9.

68. Longo F, Lynn J, McCulloh D, Chambers E. 1986. Correlative ultrastructural and electrophysiological studies of sperm-egg interactions of the sea urchin, *Lytechinus variegatus*. Developmental Biology 118:155-166.

69. Zulliger D, Lessios H. 2010. Phylogenetic relationships in the genus *Astropecten* Gray (Paxillosida: Astropectinidae) on a global scale: molecular evidence for morphological convergence, species-complexes and possible cryptic speciation. Zootaxa:1-19.

70. Byrne M, Hart M, Cerra A, Cisternas P. 2003. Reproduction and larval morphology of broadcasting and viviparous species in the *Cryptasterina* species complex. Biological Bulletin 205:285-294.

71. Hart M, Byrne M, Smith M. 1997. Molecular phylogenetic analysis of life-history evolution in asterinid starfish. Evolution 51:1848-1861.

72. Emlet R. 2016. The nonfeeding auricularia of *Holothuria mexicana* (Echinodermata, Holothuroidea). Invertebrate Biology 135:245-251.

73. Rogers A, Hamel J-F, Quetzal J, Mercier A. 2021. Unique reproductive biology of the broadcasting sea cucumber *Holothuria floridana*: facultative recruitment on adults inside nursery grounds. Invertebrate Reproduction & Development 65:141-153.

74. O’Hara T, Hugall A, Cisternas P, Boissin E, Bribiesca-Contreras G, Sellanes J, Pauly G, Byrne M. 2019. Phylogenomics, life history and morphological evolution of ophiocomid brittlestars. Molecular Phylogenetics and Evolution 130:67-80.

75. Selvakumaraswamy P, Byrne M. 2004. Metamorphosis and developmental evolution in *Ophionereis* (Echinodermata: Ophiuroidea). Marine Biology 145:87-99.

76. Collin R. 2007. Phylogenetic effects, the loss of complex characters, and the evolution of development in calyptraeid gastropods. Evolution 58:1488-1502.

77. Schneider S, Fischer A, Dorresteijn A. 1992. A morphometric comparison of dissimilar early development in sibling species of *Platynereis* (Annelida, Polychaeta). Developmental Biology 201:243-256.

78. Emlet R. 1986. Facultative planktotrophy in the tropical echinoid *Clypeaster rosaceus* (Linnaeus) and a comparison with obligate planktotrophy in *Clypeaster subdepressus* (Gray) (Clypeasteroida, Echinoidea). Journal of Experimental Marine Biology and Ecology 95:183-202.

79. Parks A, Bisgrove B, Wray G, Raff R. 1989. Direct development in the sea urchin *Phyllacanthus parvispinus* (Cidaroidea): phylogenetic history and functional modification. Biological Bulletin 177:96-109.

80. Olson R, Cameron J, Young C. 1993. Larval development (with observations on spawning) of the pencil urchin *Phyllacanthus imperialis*: a new intermediate larval form? Biological Bulletin 185:77-85.
